# Expression analysis of selected genes involved in tryptophan metabolic pathways in Egyptian children with Autism Spectrum Disorder and learning disabilities

**DOI:** 10.1038/s41598-021-86162-w

**Published:** 2021-03-25

**Authors:** Aliaa M. Higazi, Hanan M. Kamel, Emad A. Abdel-Naeem, Noha M. Abdullah, Doaa M. Mahrous, Ashraf M. Osman

**Affiliations:** 1 Clinical and Molecular Chemistry Unit, Department of Clinical and Chemical Pathology, Faculty of Medicine, Minia University, Minia, Egypt; 2 Immunology Unit, Department of Clinical and Chemical Pathology, Faculty of Medicine, Minia University, Minia, Egypt; 3 Department of Pediatrics, Faculty of Medicine, Minia University, Minia, Egypt

**Keywords:** Genetics research, Molecular medicine, Disease genetics

## Abstract

Autism Spectrum Disorder (ASD) and learning disabilities are neurodevelopmental disabilities characterized by dramatically increasing incidence rates, yet the exact etiology for these disabilities is not identified. Impairment in tryptophan metabolism has been suggested to participate in the pathogenesis of ASD, however, further validation of its involvement is required. Additionally, its role in learning disabilities is still uninvestigated. Our objective was to evaluate some aspects of tryptophan metabolism in ASD children (N = 45) compared to children with learning disabilities (N = 44) and healthy controls (N = 40) by measuring the expression levels of the *MAOA*, *HAAO* and *AADAT* genes using real-time RT-qPCR. We also aimed to correlate the expression patterns of these genes with parental ages at the time of childbirth, levels of serum iron, and vitamin D3 and zinc/copper ratio, as possible risk factors for ASD. Results demonstrated a significant decrease in the expression of the selected genes within ASD children (*p* < 0.001) relative to children with learning disabilities and healthy controls, which significantly associated with the levels of our targeted risk factors (*p* < 0.05) and negatively correlated to ASD scoring (*p* < 0.001). In conclusion, this study suggests that the expression of the *MAOA*, *HAAO* and *AADAT* genes may underpin the pathophysiology of ASD.

## Introduction

Autism Spectrum Disorder (ASD) and learning disabilities are grouped as neurodevelopmental disorders^1^. Diagnostic and Statistical Manual of Mental Disorders (DSM-5) described ASD as defects in social participation, either verbal or non-verbal difficulties in communication, and stereotyped forms of movements^2^. In 2014, a learning disability was defined as a neurological condition that interferes with an individual’s ability to store, process, or produce information according to National Adult Literacy and Learning Disabilities Center^3^. Taken together, all children with autism have at least one form of learning disability, however, not all children with learning disabilities are autistic. Therefore, it is crucial to differentiate between them. The rate of incidence of both ASD and learning disabilities is significantly increasing. It was determined that ASD is diagnosed in approximately 1 in 59 children worldwide^1^. However, the prevalence of learning disabilities is usually divided according to age group, residence, severity, and income. It was reported that up to 10% of children (between the ages of 6 to 18 years old) are distinguished as having at least one form of a learning disability such as dyslexia or dyscalculia^4^.

ASD and learning disabilities are multi-factorial and heterogeneous conditions both in their etiology and clinical presentations, and their risk genes remain largely unknown. Nowadays, investigating the etiological bases underlying these diseases becomes increasingly important due to their continuous rise in incidence over the last two decades in addition to the lack of precise prenatal screening tools, early diagnostic biomarkers, and effective treatments. As well, these disabilities impose numerous difficulties on the families of children with autism or learning abilities^5,6^.

In previous studies, it was reported that autistic children share several biochemical deficiencies and metabolic abnormalities which were considered as possible risk factors for the development of ASD such as deficiencies in maternal or autistic patients iron serum levels, vitamin D3 serum levels and zinc/copper ratios. Abnormalities in the levels of tryptophan metabolites in the peripheral blood, urine, or brain tissues were also previously associated with ASD. However, most of these studies were performed on animals or tissues rather than human blood samples. Moreover, the data collected from these studies were inconsistent or not correlated with suggested risk factors^7,8^.

The essential amino acid, L-tryptophan, is crucial for neurodevelopment. Although a small percentage of it is metabolized into the neurotransmitter, serotonin, the majority of L-tryptophan enters the kynurenine pathway to produce quinolinic acid (QA), kynurenic acid (KA), kynuramines, picolinic acid, and NAD; hence, it could play a role in regulating central nervous system (CNS) function^9^. Tryptophan metabolic pathways are regulated by an array of genes. A large-scale genetic analysis was previously performed to investigate genes involved in tryptophan metabolism in ASD and revealed a complex genetic architecture using microarray technique in animal brain tissues^10^. However, the data collected from this study have not been validated through quantitative genetic analyses. The advantage of quantitative genetic analyses is the ability to detect gene variants and pathways associated with the disease of interest, thus, it could aid in elucidating underlying biological mechanisms.

Tryptophan metabolic pathways are understudied in the context of learning disabilities and ASD. Therefore, further research is warranted. In the current study, we evaluated the expression of selected genes involved in the pathways of tryptophan metabolism in the blood samples from children with learning disabilities and ASD. These genes include monoamine oxygenase A (*MAOA*), 3-hydroxyanthranilate oxygenase (*HAAO*) and aminoadipate aminotransferase (*AADAT*). Furthermore, we correlated the expression levels of these genes with the severity of ASD and with our studied risk factors.

## Results

### Demographic and laboratory data of children within studied groups

The age of diagnosis was significantly different (*p* = 0.01) between children with learning disabilities and autism, where ASD showed to be detected earlier. We also found that ASD is more prevalent among siblings (37.8% of children with ASD compared to 4.5% of children with learning disabilities). Our data showed that the incidence of ASD compared to learning disabilities was significantly higher with advanced parental age at the time of childbirth (*p* = 0.001) (Table 1). The demographic data of autistic children according to their scoring or degree of severity is shown in Table 2.

**Table 1:** Demographic data of children within studied groups.

	GI N = 40	GII N = 44	GII**I** N = 45	*p*-values		
				GI vs GII	GI vs GIII	GII vs GIII
Age of sampling (years)						
Range	4–13	6–13	4–10	0.6	0.4	0.002*
Mean ± SD	7.6 ± 3.4	9.1 ± 2.3	6.1 ± 2.1			
Age of diagnosis (years)						
Range	-	5–10	3–5	–	-	0.01*
Mean ± SD	–	8.3 ± 0.3	4.0 ± 0.5			
Sex (%)						
Male	22(55%)	32(72.7%)	32(71.1%)	0.3	0.2	0.8
Female	18(45%)	12(27.3%)	13(28.9%)			
Siblings (%)						
No	36(90%)	42(95.5%)	28(62.2%)	0.4	0.02*	0.004*
Yes	4(10%)	2(4.5%)	17(37.8%)			
Age of mother (years)						
At time of childbirth						
Range	20–37	20–37	24–44	0.4	0.001*	0.001*
Mean ± SD	25.7 ± 4.1	26.7 ± 3.8	32.7 ± 4.8			
Age of father (years)						
At time of childbirth						
Range	25–40	26–40	29–52	0.7	0.001*	0.001*
Mean ± SD	31.2 ± 3.7	31.7 ± 3.3	38.8 ± 5.2			

GI; control group, GII; learning disabilities, GIII; autism spectrum disorder, N; number, SD; standard deviation, *p-value < 0.05 = significant.

**Table 2:** Demographic data of children within studied ASD subgroups.

	GIIIa N = 21	GIIIb N = 12	GIIIc N = 12	*p*-values		
				GIIIa vs GIIIb	GIIIa vs GIIIc	GIIIb vs GIIIc
Age of sampling (years)						
Range	7–10	5–9	4–6	0.053	0.001*	0.01*
Mean ± SD	8.5 ± 1.1	7.2 ± 2.1	5.5 ± 1.7			
Age of diagnosis (years)						
Range	4–5	4–5	3–5	0.6	0.003*	0.03*
Mean ± SD	4.7 ± 0.5	4.1 ± 0.7	3.1 ± 0.9			
Sex (%)						
Male	14(66.7%)	8(66.7%)	10(83.3%)	0.7	0.5	0.6
Female	7(33.3%)	4(33.3%)	2(16.7%)			
Siblings (%)						
No	19(90.5%)	7(58.3%)	2(16.7%)	0.08	0.001*	0.09
Yes	2(9.5%)	5(41.7%)	10(83.3%)			
Age of mother (years)						
At time of childbirth						
Range	24–38	28–40	30–44	0.007*	0.001*	0.08
Mean ± SD	29.8 ± 4.1	33.9 ± 3.6	36.7 ± 4.1			
Age of father (years)						
At time of childbirth						
Range	29–43	35–47	38–52	0.002*	0.001*	0.09
Mean ± SD	35.4 ± 4.03	40.4 ± 3.7	43.3 ± 4.5			

GIIIa; mild autism spectrum disorder, GIIIb; moderate autism spectrum disorder, GIIIc; severe autism spectrum disorder, N; number, SD; standard deviation, *p-value < 0.05 = significant.

With regards to laboratory data, the current study showed a statistically significant decrease in levels of hemoglobin (*p* = 0.04), iron (*p* = 0.001) and Vitamin D3 (*p* = 0.007) in children with learning disabilities relative to healthy children, while zinc/copper ratios remained indifferent (*p* = 0.08). Similar results were obtained in children with ASD regarding hemoglobin (*p* = 0.006), iron (*p* = 0.001) and Vitamin D3 (*p* < 0.001) levels, yet a statistically significant decrease in zinc/copper ratio (*p* < 0.001) was also observed. In addition, our data revealed a statistically significant decrease in levels of hemoglobin (*p* = 0.049), Vitamin D3 (*p* = 0.02) and zinc/copper ratio (*p* = 0.005), but not with serum iron levels (*p* = 0.3), in autistic children compared to those with learning disabilities (Table 3). The laboratory data of ASD subgroups are reported in Table 4.

**Table 3:** Comparison between studied groups regarding laboratory data.

	GI N = 40	GII N = 44	GIII N = 45	*p*-values		
				GI vs GII	GI vs GIII	GII vs GIII
Hb (g/dL)						
Range	12.8–15.0	11.9–14.4	10.9–13.3	0.04*	0.006*	0.049*
Mean ± SD	13.9 ± 0.6	12.8 ± 0.6	12.1 ± 0.6			
Iron (μg/dL)						
Range	57–121	38–103	32–132	0.001*	0.001*	0.3
Mean ± SD	80.6 ± 17.1	62.7 ± 13.1	58.3 ± 18.02			
Vit D_3_ (ng/mL)						
Range	39–67	7.1–46	6.1–38.6	0.007*	< 0.001*	0.02*
Mean ± SD	51.8 ± 7.5	17.7 ± 8.4	10.5 ± 5.6			
Zn/Cu ratio						
Range	0.77–0.99	0.73–0.91	0.51–0.75	0.08	< 0.001*	0.005*
Mean ± SD	0.87 ± 0.1	0.75 ± 0.09	0.53 ± 0.17			

GI; control group, GII; learning disabilities, GIII; autism spectrum disorder, N; number, SD; standard deviation, Hb; hemoglobin, Vit D_3_; Vitamin D_3,_ Zn/Cu; Zinc/Copper, *p-value < 0.05 = significant.

**Table 4:** Levels of risk factors in children within ASD subgroups.

	GIIIa N = 21	GIIIb N = 12	GIIIc N = 12	*p*-values		
				GIIIa vs GIIIb	GIIIa vs GIIIc	GIIIb vs GIIIc
Hb (g/dL)						
Range	11.2–13	10.9–13.3	10.9–12.9	0.01*	0.001*	0.046*
Mean ± SD	12.1 ± 0.5	11.3 ± 1.0	11.0 ± 0.9			
Iron (μg/dL)						
Range	50–132	40–104	32–73	0.001*	0.001*	0.3
Mean ± SD	66.1 ± 14.4	56.3 ± 17.5	54.1 ± 8.3			
Vit D_3_ (ng/mL)						
Range	6.4–38.6	6.7–20.8	6.1–7.6	0.003*	0.002*	0.02*
Mean ± SD	13.2 ± 6.8	9.2 ± 3.8	6.9 ± 0.4			
Zn/Cu ratio						
Range	0.66–0.75	0.59–0.65	0.51–0.55	0.002*	0.001*	0.04*
Mean ± SD	0.71 ± 0.15	0.62 ± 0.10	0.53 ± 0.03			

GIIIa; mild autism spectrum disorder, GIIIb; moderate autism spectrum disorder, GIIIc; severe autism spectrum disorder, N; number, SD; standard deviation, Hb; hemoglobin, Vit D_3_; Vitamin D_3,_ Zn/Cu; Zinc/Copper, *p-value < 0.05 = significant.

### Relative expression levels of selected genes within studied groups plus their correlations with laboratory data and with each other's

**Figure 1:**
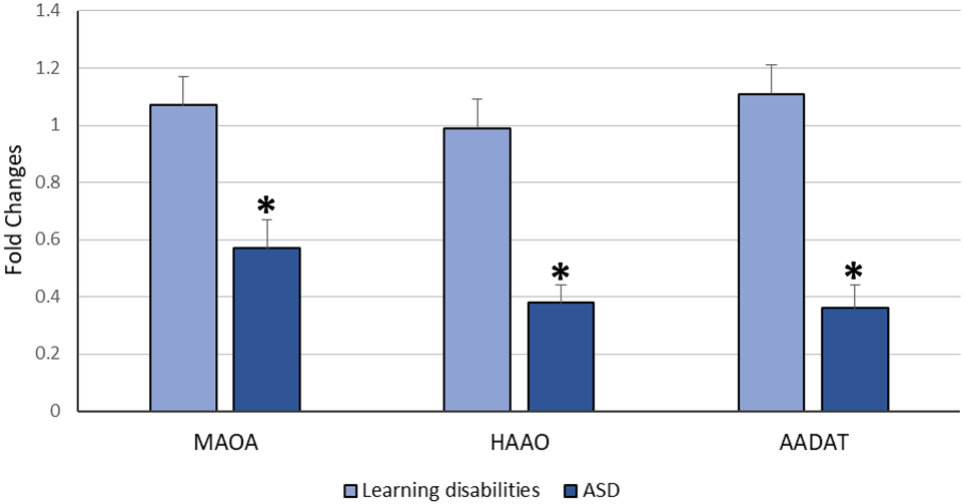
Mean fold change of *MAOA*, *HAAO* & *AADAT* genes expression in diseased groups. The mean fold changes of these genes’ expression were calculated as fold change from their expression in the control group. *p-value < 0.05 = significant. ASD; Autism Spectrum Disorder, *MAOA*; monoamine oxygenase A, *HAAO*; 3-hydroxy anthranilate oxygenase, *AADAT*; aminoadipate aminotransferase.

**Figure 2:**
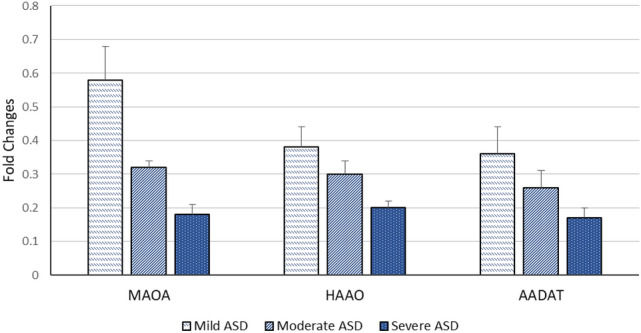
Mean fold change of *MAOA*, *HAAO* & *AADAT* genes expression according to ASD severity. The mean fold changes of these genes’ expression were calculated as fold change from their expression in controls. ASD; Autism Spectrum Disorder, *MAOA*; monoamine oxygenase A, *HAAO*; 3-hydroxy anthranilate oxygenase, *AADAT*; aminoadipate aminotransferase.

**Figure 3:**
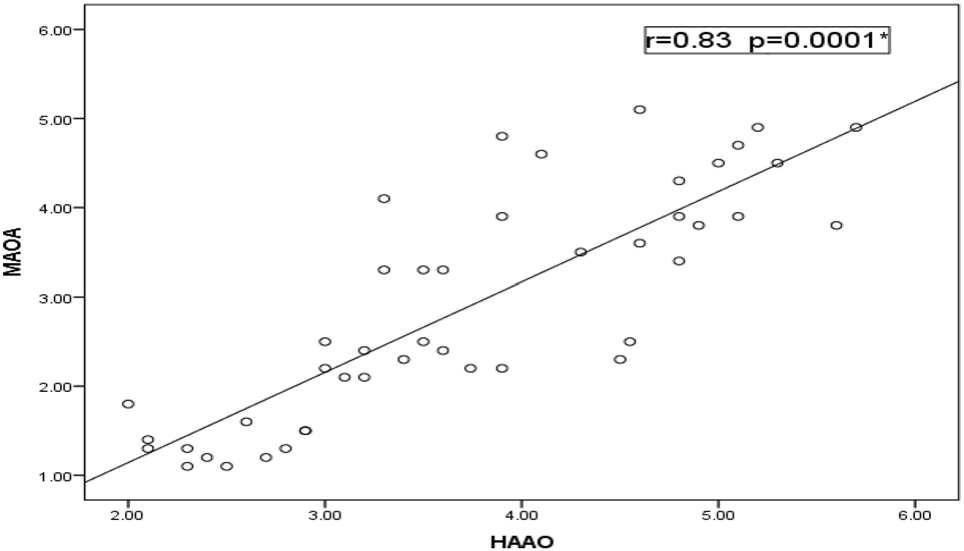
Correlation between relative expression levels of *MAOA* & *HAAO* genes among ASD. There was a statistically significant strong positive correlation between *MAOA* and *HAAO* genes’ expression (r = 0.83 & *p* = 0.0001). *MAOA*, monoamine oxygenase A; *HAAO*, 3-hydroxy anthranilate oxygenase.

**Figure 4:**
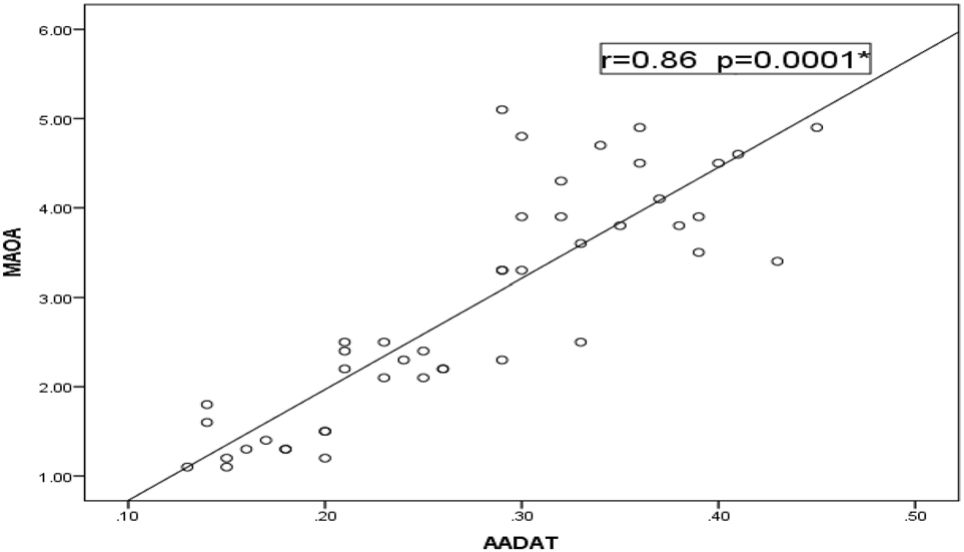
Correlation between relative expression levels of *MAOA* & *AADAT* genes among ASD. There was a statistically significant strong positive correlation between *MAOA*, *AADAT* genes’ expression (r = 0.86 & *p* = 0.0001). *MAOA*, monoamine oxygenase A; *AADAT*, aminoadipate aminotransferase.

**Figure 5:**
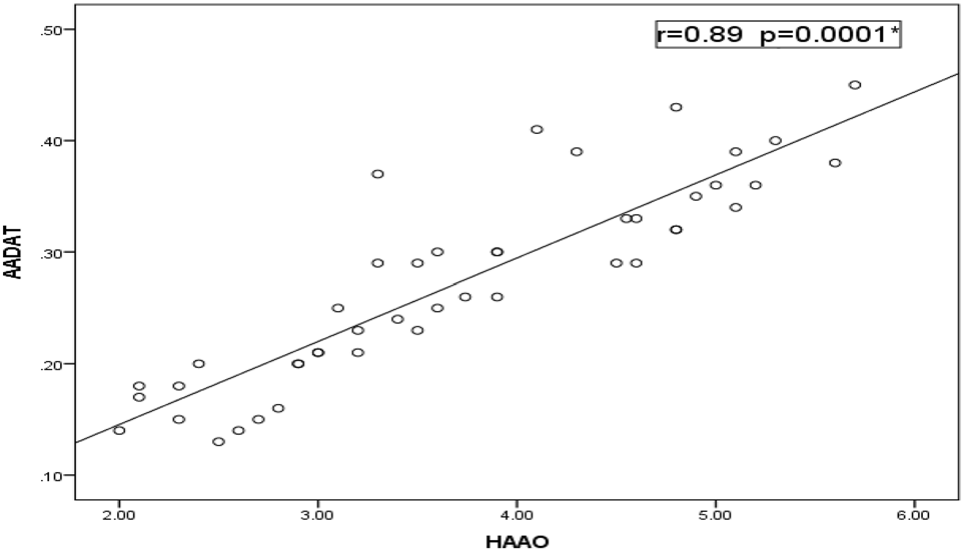
Correlation between relative expression levels of *AADAT* & *HAAO* genes among ASD. There was a statistically significant strong positive correlation between *HAAO* and *AADAT* genes’ expression (r = 0.89 & *p* = 0.0001). *AADAT*, aminoadipate aminotransferase; *HAAO*, 3-hydroxy anthranilate oxygenase.

Relative to healthy control children, our data detected a significant decrease in the expression levels of *MAAO*, *HAAO* and *AADAT* genes among children with ASD, but indifferent expression levels in children with learning disabilities relative to both controls and ASD children (Fig. 1). Regarding the severity of autism, the significant decrease in expression levels of our selected genes is associated with an increased autism score (Fig. 2). According to the laboratory data of each child, there were significant positive correlations between levels of iron and *MAOA* gene expression (r = 0.26 & *p* = 0.048), and between levels of iron and *HAAO* gene expression (r = 0.33 & *p* = 0.03) and a weak positive correlation with *AADAT* gene expression (r = 0.23, *p* = 0.01). Furthermore, there were fair positive correlations between the levels of vitamin D3 and *MAOA* (r = 0.41 & *p* = 0.005), *HAAO* (r = 0.39 & *p* = 0.007) and *AADAT* (r = 0.48 & *p* = 0.001) genes expression. Regarding zinc/copper ratios, there was a significant, moderate positive correlation with *MAOA* gene expression (r = 0.53 & *p* = 0.04), a weak positive correlation with *HAAO* gene expression (r = 0.21 & *p* = 0.003) and a fair positive correlation with *AADAT* gene expression (r = 0.35 & *p* = 0.01) (Table 5). Finally, our data reported that decreased expression levels of one of these genes are associated with a reduction in the expression level of the other two genes (Figs. 3, 4, 5).

**Table 5:** Correlation between relative gene expression & laboratory data of diseased groups.

	Learning disabilities N = 44			ASD N = 45		
	*MAOA*	*HAAO*	*AADAT*	*MAOA*	*HAAO*	*AADAT*
Hb						
r	0.27	0.02	0.20	0.05	0.11	0.05
*p*-value	0.2	0.9	0.2	0.06	0.4	0.07
Iron						
r	0.08	0.15	0.38	0.26	0.33	0.23
*p*-value	0.6	0.4	0.07	0.048*	0.03*	0.01*
Vit D_3_						
r	0.29	− 0.02	0.21	0.41	0.39	0.48
*p*-value	0.1	0.8	0.3	0.005*	0.007*	0.001*
Zn/Cu ratio						
r	0.25	0.35	0.18	0.53	0.21	0.35
*p*-value	0.06	0.5	0.07	0.04*	0.003*	0.01*

r = 0.75–1 (strong correlation), r = 0.5–0.74 (moderate correlation),r = 0.25–0.49 (fair correlation), r = 0.1–0.24 (weak correlation), *p-value < 0.05 = significant, ASD; Autism Spectrum Disorder, N; number, *MAOA*; monoamine oxygenase A, *HAAO*; 3-hydroxy anthranilate oxygenase, *AADAT*; aminoadipate aminotransferase, Hb; hemoglobin, Vit D_3_; vitamin D_3_, Zn/Cu; Zinc/Copper.

### Correlation between ASD severity and parental ages, laboratory data along with expression levels of selected genes

In this study, there were statistically significant, positive correlations between parental age at the time of childbirth and ASD severity (*p* = 0.042 for mothers’ age, 0.037 for fathers’ age). As well, we estimated that Vitamin D3 levels and zinc/copper ratios significantly decreased with increased ASD severity (r = − 0.473 & r = − 0.333 respectively & *p* = 0.001). Levels of *MAOA*, *HAAO* and *AADAT* genes expression in autistic children showed a significantly strong, negative correlation with the degree of ASD severity (r = − 0.932, r = − 0.83, r = − 0.891 respectively & *p* < 0.001) (Table 6).

**Table 6:** Correlation between ASD severity along with parental age, laboratory data of children and their relative genes expression.

ASD severity	Age of mothers at time of childbirth	Age of fathers at time of childbirth	Hb	Iron	Vit D_3_	Zn/Cu ratio	*MAOA*	*HAAO*	*AADAT*
r	0.23	0.31	− 0.097	− 0.204	− 0.473	− 0.333	− 0.932	− 0.830	− 0.891
*p*-values	0.042*	0.037*	0.528	0.179	0.001*	0.001*	< 0.001*	< 0.001*	< 0.001*

r = 0.75–1 (strong correlation), r = 0.5–0.74 (moderate correlation),r = 0.25–0.49 (fair correlation), r = 0.1–0.24 (weak correlation), *p-value < 0.05 = significant, ASD; Autism Spectrum Disorder, Hb; hemoglobin, Vit D_3_; vitamin D_3_, Zn/Cu; Zinc/Copper, *MAOA*; monoamine oxygenase A, *HAAO*; 3-hydroxy anthranilate oxygenase, *AADAT*; aminoadipate aminotransferase.

### Regression analysis regarding the expression of selected genes and different co-variants in relation to ASD diagnosis along with between targeted genes and different co-variants

We analyzed the regression association between our targeted genes expression along with the sex of included subjects and their ages at the time of sampling and diagnosis in relation to disease diagnosis. Based on coefficient values and 95% CI (coefficient intervals), the results in the current study revealed that the diagnosis of ASD is dependent on the expression of *MAOA*, *HAAO* and *AADAT* genes (*p*-values < 0.001 for all) but not on other included co-variants (*p*-values > 0.05 for all) (Table 7). Also, we investigated the associations of candidate genes expression with different co-variants including our subjects’ sex, the age of sampling as well as the age of diagnosis via linear regression analysis. However, our data revealed that the expression of *MAOA*, *HAAO* and *AADAT* genes are not associated with our included co-variants (*p*-values > 0.05 for all). These data mean that the expression levels of our three selected genes are not dependent on the levels of these co-variants. The detailed statistical regression measures are shown in Table 8.

**Table 7:** Regression analysis for the association between sex, age of sampling, age of diagnosis and targeted genes expressions in relation to disease diagnosis.

	Coefficient	Std. Error	95%CI	*p*-value
Sex	1.6206	0.1622	1.2979, 1.9430	0.254
Age of sampling	1.8776	0.1644	1.55, 2.2	0.231
Age of diagnosis	3.871	0.11	3.851, 4.091	0.052
*MAOA*	1.7774	0.07333	1.63, 1.92	< 0.001*
*HAAO*	2.2992	0.06593	2.17, 2.43	< 0.001*
*AADAT*	2.2209	0.06065	2.1, 2.3	< 0.001*

Std. Error; standard error, 95% CI; 95% confidence interval, *MAOA*; monoamine oxygenase A, *HAAO*; 3-hydroxy anthranilate oxygenase, *AADAT*; aminoadipate aminotransferase, *p-value < 0.05 = significant.

**Table 8:** Regression analysis for the association between targeted genes expressions and sex, age of sampling along with age of diagnosis.

	Co-variants	Coefficient	Std. Error	95% CI	*p*-value
*MAOA*	Sex	4.481	1.336	1.824, 7.138	0.865
	Age of sampling	5.594	1.389	2.833, 8.355	0.497
	Age of diagnosis	0.962	0.472	0.02, 1.904	0.055
*HAAO*	Sex	4.568	1.079	2.423, 6.712	0.073
	Age of sampling	5.661	1.142	3.390, 7.932	0.488
	Age of diagnosis	0.947	0.476	− 0.003, 1.897	0.051
*AADAT*	Sex	0.354	0.096	0.162, 0.545	0.081
	Age of sampling	0.432	0.102	0.230, 0.634	0.396
	Age of diagnosis	− 0.720	0.108	− 0.936, − 0.504	0.051

Std. Error; standard error, 95% CI; 95% confidence interval, *MAOA*; monoamine oxygenase A, *HAAO*; 3-hydroxy anthranilate oxygenase, *AADAT*; aminoadipate aminotransferase.

## Discussion

ASD is a major health problem worldwide^11^. Learning disability is also very common; the American Academy of Child and Adolescent Psychiatry reported that at least 1 in 10 school children are affected by learning disorders and are receiving special education services^12^. The possible causes of both ASD and learning disabilities are still uncertain, but researchers have found a range of risk factors that may be present from birth and tend to run in families.

In the current study, siblings represented only 4.5% of our included learning disability children. In disagreement with our data but in keeping with most published literature, siblings of an Indian background with dyslexia were shown to have significant problems in selective attention^13^. This could be attributed to a difference in the used methodologies. On the other hand, 45 children with ASD were included in our study, and 17 of them were siblings. Based on studies of families, the recurrence risk of ASD among siblings was estimated at anywhere between 3 and 10%^14^. In a study performed by Miller et al., researchers found an elevated rate of ASD recurrence in later-born siblings^15^. Likewise, Ozonoff et al. and an expansion study in 2015 indicated a 19.5% recurrence rate in ASD siblings with some variability in estimates across 15 different study sites. Thus, the recurrence risk for a child depends on both genetic and environmental factors^16,17^. Similarly, it was revealed that not all diagnosed cases of ASD and learning disabilities are genetically based, but environmental factors could play a role^15^. However, most of the previous studies were focused on either the genetic view of these disorders or their environmental risk factors with less emphasis on their correlation to each other^18,19^. In the current study, we investigated some genetic aspects related to tryptophan metabolism pathways within ASD and learning disability children in association with a set of possible environmental risk factors containing parental ages, serum levels of iron, vitamin D3 as well as zinc/copper ratios.

Our results show that older ages of fathers and mothers at the time of childbirth of their ASD and learning disability children were led to a significantly higher incidence of children born with ASD and learning disability compared to the control group. Furthermore, we found that children born to parents of advanced ages are associated with a higher risk of developing ASD, which agrees with previous studies^20–28^. Sandin et al.^27^ recorded an increased incidence of ASD when there are increased differences between parental ages and not only because of advanced paternal or maternal ages. Similarly, the association between learning disability and advanced parental ages was reported before^28^. Altogether, our data present an evidence for the possible incorporation of accumulated parental age-related genetic mutations and epigenetic modifications in the pathogenesis of these neurodevelopmental disorders.

The present study displays a significant decrease in the levels of serum iron and lower hemoglobin concentrations in children with ASD and learning disabilities in comparison to healthy controls. These findings were similar to those recorded in many previous studies^29–33^. Moreover, it was reported that maternal intake of iron supplements reduces their risk of having an autistic child^34^. Many regions in the brain contain the highest concentrations of iron in the body because iron is crucial for the myelination of neurons and the synthesis of neurotransmitters. Additionally, the brain is using 20% of basal body oxygen levels to maintain mitochondrial ATP production and brain iron deficiency has been shown to damage mitochondrial DNA and change mitochondrial morphology and function^29^.

Our study showed a significant decrease in the levels of vitamin D in both children with ASD and learning disabilities. It was revealed that reduction in the brain and serum levels of vitamin D could play a role in the development of the unordinary social behaviours that occur in a number of psychological conditions, including autism^35^. Likewise, early childhood and gestational vitamin D deficiencies were demonstrated within autistic children and their mothers in many previous studies^36–39^. In accordance, children of women who had higher levels of vitamin D were estimated to have a lower risk of autism. Additionally, it was shown that vitamin D supplements may improve autism symptoms in autistic children who have vitamin D deficiency or insufficiency^40,41^. The overall prevalence of vitamin D deficiency was 77.3% in the learning disabilities group and 39.6% in the control group as stated in the study by Cebulla^42^. Similar results were concluded in Frighi and coauthors study^43^. On top, we found a significant correlation between levels of vitamin D and severity of autism which agrees with the study conducted by El-Ansary et al.^44^. The association between vitamin D deficiency and neurodevelopmental disorders was claimed mainly to its role in immune regulation. Alongside this association, certain polymorphisms in vitamin D receptor genes were distinguished as possible risk factors for the development of ASD^45,46^.

Zinc is utilized in the human body for normal growth and development from the time of intrauterine development^47^. Its deficiency may cause neuropsychological problems such as emotional instability, irritability, and depression. As well, it was established that copper toxicity severely affects the brain^48,49^. The current research revealed a significant decrease in zinc/copper ratios in ASD cases when compared to both children with learning disabilities and the control group (*p* = 0.005 & < 0.001 respectively). It was reported that children with ASD appear to be at risk for zinc deficiency and copper toxicity and thus often have low zinc/copper ratios. Furthermore, a number of studies considered this decreased ratio as a chemical marker for ASD. Additionally, our data stated a significant fair negative correlation between this ratio and the scoring of ASD (r = − 0.333 & *p* = 0.001) which agreed with a number of previous studies^50–52^.

Tryptophan plays vital structural and functional roles in neurodevelopment as it is involved in the synthesis of cell membrane proteins as well as being a precursor for the neurotransmitter serotonin, the neurohormone melatonin, niacin (which is also known as vitamin B3), as well as auxins (a class of phytohormones). Also, tryptophan is mainly metabolized through the kynurenine pathway which is a source for NAD^+^^53^. The metabolomics in the urine of young children with ASD revealed alterations in a number of metabolic pathways containing tryptophan metabolisms. Dysregulation in tryptophan metabolism was found to cause an increase in QA and a decrease in both KA and melatonin. These metabolic abnormalities could be responsible for seizures, sleep disorders, gastrointestinal disturbances, and some other symptoms related to ASD^54^. Besides, it was discovered that maternal inflammations lead to altered placental tryptophan metabolism by inducing transcriptional and translational cascades in the placenta, which in turn cause abnormality in human fetus neurochemistry and neurodevelopment^55,56^. Therefore, impairment in tryptophan metabolic pathways was strongly suggested as a possible genetic risk factor that influences the development of ASD which needs further investigations. Furthermore, its role in learning disabilities is not inspected yet.

In the current study, we measured the expression levels of three genes involved in tryptophan metabolic pathways, which are *MAOA* (serotonin pathway) along with two genes within the kynurenine pathway (*HAAO* and *AADAT*) in both children with ASD and learning disabilities. We found disturbances through detection of lower expression levels of these genes in patients with ASD only, which is correlated with the severity of autism. Boccuto et al. used lymphoblastoid cell lines from patients with neurodevelopmental disorders including ASD. They analyzed the metabolic profile of these cells. Their results showed a significant difference in the expression levels of *MAOA, HAAO, AADAT, SLC7A5, SLC7A8, TPH2* and *WARS2* genes in cases with neurodevelopmental disorders when compared to controls while the relative expression level of *QPRT* gene showed no significant difference. These data were analyzed through a microarray study which in turn needs more validations^10^. The results of this study agree with our results regarding *MAOA, HAAO* and *AADAT* genes.

MAOA enzyme is the primary metabolizing enzyme for monoamine neurotransmitters including serotonin. Thus, it is a key regulator for brain function. Individuals who had loss of function mutations in *MAOA* gene and a deficient MAOA activity showed increased levels of serotonin^57–62^ which appear to take part in the pathogenesis of ASD through a vast of neuro-immune responses^63^. It was found that dysregulation in the body levels of serotonin is associated with an increased incidence of abnormal behaviours which mimic many ASD-associated ones^64,65^. Our findings showed a significant decrease in the expression of the *MAOA* gene which is correlated with the degree of autism severity. Likewise, Gu et al. measured MAOA activity from post-mortem tissues of the cerebellum and the frontal cortex from subjects with autism. They found that the activity of MAOA in the cerebellum is lowered by 20.6% while in the frontal cortex is lowered by 27.8%^66^.

It has been reported that altered peripheral blood or cerebral levels of either QA or KA may participate in the pathogenesis of neurologic disorders incorporating many ASD characteristics^67,68^. The *HAAO* gene is involved in the kynurenine pathway of tryptophan metabolism. It encodes an enzyme that catalyzes the synthesis of QA which is a precursor of NAD^53^. NAD is a precursor of NADH, an energy carrier for the mitochondria electron transport chain. HAAO is an excitotoxin as well that has been suggested to be involved in disorders associated with altered tissue levels of QA. According to our study, there is a decrease in *HAAO* gene expression in subjects with ASD which may lead to a reduction in NAD synthesis and an impairment in mitochondrial function. It was reported that a loss of function mutation of the maternal *HAAO* gene causes NAD deficiency and congenital anomalies^53^. Finally, the *AADAT* gene encodes an enzyme that catalyzes the process of kynurenine transamination to KA^69^. A new missense mutation in *AADAT* gene was discovered by Li et al. which may provide a potential molecular base for ASD in families^70^.

## Subjects and methods

This study was carried out at the Clinical and Chemical Pathology along with Pediatrics Departments, Faculty of Medicine, Minia University, Egypt. It was conducted in accordance with the ethical guidelines of both Declaration of Helsinki and the International Conference on Harmonization Good Clinical Practice. One hundred twenty-nine children were included. They were classified into three main groups. Group I was containing 40 apparently healthy control children in addition to patients’ groups (Group II and III). Patients’ groups contained 89 children selected from patients who regularly followed up at the Neuropsychiatric Clinic, Pediatric Hospital, Minia University, Egypt and from Kayan charity for education and rehabilitation of autistic children, Minia Branch, Egypt. The patients in group II (N = 44) involved children with learning disabilities while group III children (N = 45) are those with ASD. The ASD group was further subdivided according to the severity of autism into group IIIa with mild autism, group IIIb with moderate autism and group IIIc with severe autism. The diagnosis of learning disabilities was performed through intelligence tests (IQ tests), achievement tests, visual-motor integration tests and language tests^71^, while ASD children were diagnosed as defined by DSM-5 criteria. The severity of ASD was characterized according to Childhood Autism Rating Scale (CARS)^72^. Diagnosis of all patients was made by proficient specialists in this field. Informed consent was signed by parents of all participants and study approval was obtained from the review board of the Minia Faculty of Medicine, Minia University, Egypt. Patients with mental retardation, psychological disorders e.g. schizophrenia, psychopathological disease e.g. brain tumour, type I IDDM or IQ-Achievement discrepancy were excluded.

An initial evaluation for all participants incorporated a detailed history, including prenatal, natal and post-natal histories. All children were subjected to clinical examination including psychological examination according to DSM-5 criteria and radiological examination including brain computerized tomography (CT). Venous blood samples were withdrawn from all subjects for analysis of complete blood counts (CBCs) via automated cell counter Celltac ES (Nihon Kohden Europe) plus liver and renal function tests by automated chemistry auto-analyzer system Konelab 20i (Thermo Electron Incorporation, Vantaa, Finland) to exclude other diseases. As well, serum levels of iron, zinc and copper were assessed by the direct colorimetric method using kits from Spectrum Diagnostics (Cairo, Egypt) for iron, Quimica Clinica Aplicada S.A. (Amposta, SPAIN) for zinc and JaICA, Nikken SEIL Co. (Shizuoka, Japan) for copper. The colour absorbance was measured via Microlab 300 analyzer (ELITechGroup Clinical Systems, France). The Zn/Cu ratio was calculated. Furthermore, serum levels of vitamin D3 were measured via Enzyme-Linked Fluorescent Assay technique (ELFA) using (Mini Vidas, Biomerieux, France).

Subjects’ whole blood samples were used to quantify the expression levels of *MAOA, HAAO, AADAT* genes after RNA extraction by Gene JET RNA Purification Kit from Thermo Scientific (Waltham, Massachusetts, United States) according to manufacture instructions. RNA was further reverse transcribed to cDNA using High Capacity cDNA reverse transcription kit from Applied Biosystems, Thermo Fisher Scientific (Waltham, Massachusetts, United States). Real-time PCR was performed using TaqMan Gene Expression Assays Kit supplied by Applied Biosystems, Thermo Fisher Scientific (Waltham, Massachusetts, United States). The thermal protocol was run on a DT lite Real-Time PCR System (DNA technology, Moscow, Russia). The amounts of expression for *MAOA, HAAO* and *AADAT* genes were made relative to the expression of the housekeeping gene Beta-actin and calculated using the threshold cycle (Ct) method.

The different real-time PCR forward and reverse primers were designed as follows:

**Table:** 

	Forward Primers	Reverse Primers
*h MAOA*	5′-ACA GCC TGA CCG TGG AGA AG-3′	5′-GAA CGG ACG CTC CAT TCG GA-3′
*h HAAO*	5′-ACACCGGGATGTGGTCATTC-3′	5′-ATAGTACCTGAGCCCATCTAGC-3′
*h AADAT*	5′-GGCTGGTGGCTTACCAAATC-3′	5′-ACTCGGAGAATACTGAAGTGCTC-3′
*h Β-actin*	5′-CATGTACGTTGCTATCCAGGC-3′	5′-CTCCTTAATGTCACGCACGAT-3′

## Statistical analyses

Statistical Package for Social Sciences (SPSS) program software version 24 (SPSS Inc., Chicago, IL, USA) was used. Descriptive statistics for parametric quantitative data were presented as mean ± standard deviation (SD) and range, whereas categorical data were presented as number and percentage. Sample t-test was used for analyses of parametric data between two groups. Analysis of variance (ANOVA) of regression and coefficient analysis along with 95% CI for the coefficient estimates were used to demonstrate the significance of associations within linear regression analysis. A *p*-value ≤ 0.05 was considered statistically significant. Expression levels were presented as fold-change relative to the healthy control group. Correlations between two quantitative variables were done by Pearson's correlation coefficient. The correlation coefficient ranges were considered weak if r = 0–0.24, fair if r = 0.25–0.49, moderate if r = 0.5–0.74 and strong if r = 0.75–1. Figures were assembled using Excel office 10.

## Conclusion

Collectively, our study detected disturbances in tryptophan metabolism by measuring the expression levels of three genes involved in tryptophan metabolic pathways. We found a decrease in the expression levels of *MAOA*, *HAAO and AADAT* genes in ASD only. Learning disability children had no impairment in the metabolism of tryptophan in relation to these three genes expression patterns. Accordingly, these genes can be used for early diagnosis of autism and to differentiate between children with mild autism and children with learning disabilities which may be more difficult to clinically tell apart. The current study supported the previous work which claimed older maternal and/or paternal ages in addition to iron deficiency anemia along with vitamin D deficiency as potential environmental risk factors for the predisposition of ASD and learning disabilities. To validate these genes as markers, future studies on larger sample size and multi-central studies are required. Similar studies regarding other genes involved in tryptophan metabolism could help in more understanding of the pathogenesis and progression of autism and finding new biomarkers. Screening of pregnant mothers should be performed and should include the measurement of maternal levels of our studied chemical risk factors including maternal hemoglobin, iron and vitamin D levels. As well, the maternal expression levels of our selected genes could be suggested in prospective studies to facilitate gene therapy studies and trials for treating and improving signs and symptoms of ASD or for prenatal diagnosis and management. These findings help us to better understand and shed new light on the capable risk factors and their associations with some possible genetic etiology for the pathogenesis of ASD and learning disabilities.
